# Acute Kidney Injury after Congenital Heart Disease Surgery: A Single-Center Experience in a Low- to Middle-Income Country

**DOI:** 10.7759/cureus.7727

**Published:** 2020-04-18

**Authors:** Fatima Ali, Misha Khalid Khan, Bilal Mirza, Sonia Qureshi, Qalab Abbas

**Affiliations:** 1 Pediatrics and Child Health, Aga Khan University Hospital, Karachi, PAK

**Keywords:** congenital heart surgery, kdigo, cardiopulmonary bypass time, acute kidney injury

## Abstract

Background

Acute kidney injury (AKI) is a commonly recognized clinical problem after congenital heart disease (CHD) surgery. Increased perioperative morbidity, development of chronic kidney disease, and increased mortality are the major concerns. We investigated frequency, risk factors, and outcomes of AKI after CHD surgery at our hospital.

Methods

This study was a retrospective analytic review conducted from January 2013 to October 2016 on patients aged between 1 month and 45 years who underwent cardiopulmonary bypass (CPB) for CHD surgery. The modified Kidney Disease Improving Global Outcomes criteria based on serum creatinine value was adopted to diagnose AKI. We assessed AKI frequency and its staging, and outcomes as AKI resolution, length of stay, and mortality. Stages II and III (plasma creatinine level two or more times the baseline) were labeled as severe AKI. Univariate and multivariate logistic regression analyses were conducted, and results were reported as mean with standard deviation and as frequencies with percentage. Odds ratios (ORs) with 95% confidence intervals (CIs) were reported for factors associated with the development of AKI.

Results

Of the 840 patients who underwent CHD surgery, 237 (28%) developed AKI. AKI stages II1 and III were seen in 101 (42%) and 103 (43%) patients, respectively. Prolonged CPB time > 120 minutes (adjusted OR [AOR]: 1.87; 95% CI: 1.22-2.88; p = 0.004) and hemoglobin > 16 gm/dL (AOR: 1.80; 95% CI: 1.16-2.78; p = 0.008) were associated with the development of AKI on multivariate analysis. AKI resolved spontaneously in 222 (94%) patients, and 10 (4%) patients who developed AKI died.

Conclusions

Most patients with AKI showed spontaneous resolution. Prolonged CPB time and increased hemoglobin were found to be significant risk factors. Our study found spontaneous resolution of AKI in most cases. However, preplanning and careful monitoring in patients with expected prolonged CPB time and increased baseline hemoglobin can prevent and identify AKI at an early stage.

## Introduction

Acute kidney injury (AKI) is a common illness after congenital heart disease (CHD) surgery. The reported incidence of AKI in this population is high, ranging from 30% to 52%, with the highest incidence in neonates (60%) post-CHD surgery [1-3]. Concerns are related to not only increased risk of in-hospital mortality but also increased risk of morbidities leading to chronic renal failure, prolonged mechanical ventilation, prolonged stay in the intensive care unit, and resultant high cost of care [4]. Risk factors associated with AKI include prolonged cardiopulmonary bypass (CPB) time, complexity of surgical repair, degree of hypothermia, circulatory arrest, and postoperative low cardiac output syndromes [2-4]. A major advancement in the field of AKI research has been the development of standardized, staged definitions of AKI that allow for comparison of incidence and outcomes across studies.

AKI has been defined using various classifications including Pediatric Risk, Injury, Failure, Loss, End-Stage Renal Disease (RIFLE) criteria, Kidney Disease Improving Global Outcomes (KDIGO) criteria, and Acute Kidney Injury Network (AKIN) [5,6-9]. Luo et al. in their prospective study concluded that KDIGO is better than RIFLE and as good as AKIN [8]. Patients with AKI diagnosed by KDIGO but missed by RIFLE or AKIN had higher mortality than those without AKI, according to KDIGO [2,10,11]. The m-KDIGO is the revised form adapted to prevent noise and bias toward classifying critically ill young infants and neonates in terms of the presence of AKI. It uses a minimum serum creatinine of >0.5 mg/dL to successfully identify AKI [8,9].

Pediatric CHD services are in the beginning stage in Pakistan. There is a scarcity of resources for managing these complex patients. Many CHDs are left unrepaired on time, and there is often delay in diagnosis and surgery of these patients. The objective of our study was to determine the frequency, risk factors, and outcomes of AKI in the immediate postoperative period after CHD surgery. It will not only help in understanding the burden of this morbidity but would also be able to modify some of these factors, reduce cost, and provide preemptive management of at-risk groups, leading to better outcomes.

## Materials and methods

A retrospective analytic review was conducted at Aga Khan University Hospital (AKUH) after approval from the hospital’s ethical review committee; exemption from full review and consent was granted because of the retrospective nature of the study. AKUH is a 700-bed private tertiary care referral center for specialized adult and pediatric care, where approximately 250 CHD surgeries are performed annually. It has a four-bed pediatric cardiac intensive care unit (PCICU) staffed with a full-time intensivist and round-the-clock coverage. All patients undergoing congenital heart surgery are admitted to the PCICU. All three modalities of renal replacement therapy (RRT) are available here, including peritoneal dialysis, hemodialysis, and continuous RRT.

A medical record review was conducted from January 2013 to October 2016 of all patients who underwent CHD surgery on CPB. Patient age ranged from 1 month to 45 years. This wider age range was considered because the burden of late-presenting adult CHD in low- to middle-income countries is high, and these patients thus comprise a substantial percentage of CHD surgeries at our center.

Patients who had existing renal dysfunction (already on RRT or with glomerular filtration rate < 60 mL/minute/1.73 m2 for more than three months), those who died within 24 hours, those older than 45 years, and those (>18 years) with diabetes mellitus and hypertension were excluded. We also excluded neonatal CHD patients in order to prevent skewed results because the first few days of creatinine level reflect maternal creatinine level followed by a fall in creatinine. However, if AKI is present, creatinine may not fall, resulting in the rise in creatinine not being detected, thus leading to fewer cases detected and secondly the criterion of a minimum serum creatinine of 0.5 mg/dL at baseline. The baseline level in some neonates may be lower than 0.5 mg/dL; thus, they will not be included in the study. Therefore, the suggested definition in this paper may result in the detection of fewer cases of neonatal AKI. Cardiac surgical procedures were graded as 1-6 by complexity based on the Risk Adjustment for Congenital Heart Surgery (RACHS-1). RACHS-1 was chosen because it is the standard score used at our center for reporting outcomes [12]. Vasoactive inotropic score (VIS) was calculated as described by Gaies et al., and a score of >20 was labeled as high VIS [13-15]. The medication profile of all patients was examined to find drugs that have nephrotoxicity as their adverse effect. Data were collected using a structured proforma and extracted from the patients’ medical records. Patients were identified from the intensive care unit log-book and rechecked with a health information management system using the International Classification of Diseases coding. Data collection included demographic details (age, gender, weight, height) as well as clinical details including anatomical and physiological cardiac diagnosis, RACHS scoring, surgical procedure details, CPB and aortic cross-clamp (ACC) time, blood transfusion, inotropic support, nephrotoxic drug exposure, laboratory variables (creatinine, hemoglobin), and outcome data (staging of AKI, need of RRT, resolution of AKI and mortality).

The m-KIDGO criteria were used to define AKI and stages. AKI was defined on the basis of m-KIDGO criteria, and an increase in serum creatinine level by 0.3 mg/dL or more and a minimum serum creatinine of > 0.5 mg/dL was required at baseline to be included in the study. Stage I was defined as an increase in serum creatinine level by 0.3 mg/dL or more, up to 1.5 to 1.9 times the baseline. Stage II was defined as an increase in serum creatinine to 2.0 to 2.9 times the baseline, and Stage III was defined as an increase in serum creatinine to 3.0 times or more of the baseline.

No AKI was defined as baseline serum creatinine that did not increase by 0.3 mg/dL or more from baseline within seven days of CHD surgery. The maximum AKI stage was defined based on the increase in serum creatinine criteria. Urine output as a determinant of AKI was not used because 95% of the patients in our unit received a diuretic within 12 hours of arriving at the PCICU, which has the potential of bias and could change the results. A CPB time of >120 minutes was defined as prolonged CPB time [16]. Existing renal dysfunction was labeled in patients who had a history of chronic renal failure, those with high baseline serum creatinine (laboratory range > 1.3 mg/dL), or those who were on RRT before surgery.

Patients were followed for the first seven days of CHD surgery for the development of AKI, need for RRT, and in-hospital mortality. Complete resolution was defined as a return of serum creatinine to <50% above baseline. Similarly, an incomplete resolution was defined as serum creatinine decreased but remained >50% above baseline during their length of stay.

Statistical analysis was conducted using Stata Statistical Software, Release 12 (StataCorp LP, College Station, TX, USA). The chi-square test for categorical variables and the Student t-test for continuous variables were used. The univariate and multivariable analyses were conducted using logistic regression to assess the relationship of independent variables with the log odds of the outcome variable. Crude and adjusted odds ratios (AORs) with 95% confidence interval (CI) were reported. P-value of less than 0.05 was considered statistically significant. Results are presented as the frequency with percentages and as mean with standard deviation.

## Results

A total of 840 patients underwent CHD surgery on CPB during the study period, of which 237 (28%) developed AKI. The study population included 474 patients (calculated sample size: 237 with AKI and 237 without AKI). The mean age of the study population was 8.2 ± 9.3 years and 7.6 ± 9.5 years in the AKI and no AKI groups, respectively. Male patients comprised 151 (63.7%) of the 237 patients in the AKI group and 167 61.9%) of the 237 patients (in the no AKI group. Cyanotic CHD was present in 106 (45%) of the 237 patients in the AKI group compared with 92 (38%) of the 237 patients in the no AKI group (Table 1). AKI stage III was present in 103 patients (43%; Table 2). Of 237 patients, 222 (94%) had spontaneous resolution of AKI, while worsening of AKI was seen in 15 (6%) patients. Of the 15 patients, 5 (2%) showed resolution on follow-up, and RRT (peritoneal dialysis) was performed in three patients. Ten (4%) patients died. Complete resolution occurred in 137 patients (62%; Table 3). Also, 156 patients (66%) developed AKI in the first 24 hours, and 152 patients (64%) had peak time of AKI in the first 24 hours (Table 2). The mean duration to AKI resolution was 4.2 ± 2.4 days. The mean length of hospital stay was 8.0 ± 4.1 days in the AKI group compared with 5.1 ± 2.5 days in the no AKI group (p = 0.000). On univariate analysis, prolonged CPB time, multiple CPB time, multiple ACC time, RACHS-1, and preoperative hemoglobin > 16 gm/dL were associated with the development of AKI (Table 4). On multivariate logistic regression analysis, prolonged CPB time (>120 minutes; AOR: 1.87; 95% CI: 1.22-2.88; p = 0.004) and hemoglobin > 16 gm/dL (AOR: 1.80; 95% CI: 1.16-2.78; p = 0.008) were independently associated with AKI (Table 3).

**Table 1:** Comparison of baseline data for AKI versus non-AKI performed using Pearson’s chi-square test for categorical variables and Student’s t-test for continuous variables AKI, acute kidney injury; SD, standard deviation; RACHS, Risk Adjustment for Congenital Heart Surgery

Variable	AKI, n (%)/Mean ± SD	Non-AKI, n (%)/Mean ± SD	p-Value
Age (years)	8.2 ± 9.3	7.6 ± 9.5	0.50
Age groups			
30 days to 1 month	59 (24.8)	45 (18.9)	0.09
>1 to 5 years	69 (29.1)	100 (42.1)	
>5 to 10 years	38 (16)	41 (17.3)	
>10 to 18 years	47 (19.8)	29 (12.2)	
>18 to 30 years	16 (6.7)	15 (6.3)	
>30 to 45 years	8 (3.3)	7 (2.9)	
Weight (kg)	21.2 ± 18.7	7.6 ± 9.5	0.50
Height (cm)	107 ± 36.7	103 ± 32	0.30
Gender (male)	151 (63.7)	149 (62.8)	0.55
RACHS			
1	29 (12.3)	52 (22)	0.02
2	177 (74.6)	166 (70)	0.10
3	25 (10.5)	17 (97.2)	0.28
4	6 (2.5)	2 (0.8)	0.60
Cyanotic heart disease	106 (45)	92 (38)	0.12
Acyanotic heart disease	131 (55)	145 (61)	0.13
Single ventricle	8 (3.4)	14 (6)	0.14
Preoperative creatinine (mmol/dL)	0.3 ± 0.1	0.4 ± 0.2	0.01
Preoperative hemoglobin (mg/dL)	14.2 ± 0.2	13.5 ± 0.1	0.02
Cardiopulmonary bypass time (minutes)	111.6 ± 3.7	83.5 ± 2.3	0.00
Aortic cross-clamp time (minutes)	73.4 ± 2.5	54 ± 2.3	0.00

**Table 2:** Detail of AKI stages and outcomes AKI, acute kidney injury

Variables	N (%)/(Mean ± SD)
AKI stages	
Stage 1	33 (15)
Stage 2	101 (42)
Stage 3	103 (43)
AKI outcome	
AKI worsening	15 (6)
Resolution	5 (2)
Expired	10 (4)
Spontaneous resolution	222 (94)
Complete resolution	137 (62)
Incomplete resolution	27 (13)
No follow-up	58 (27)
Length of stay (mean ± SD)	
Case	8 ± 4.1
Control	5.1 ± 2.5
Day of AKI diagnosis	
Day 1	156 (66)
Day 2	36 (16)
Day 3	32 (14)
Day 4	4 (2)
Day 5	2 (2)
Time to peak AKI postdiagnosis	
Day 1	152 (64)
Day 2	48 (20)
Day 3	24 (10)
Day 4	4 (2)
Day 5	9 (4)
Duration of AKI	
1 day	9 (4)
2 days	56 (24)
3 days	65 (28)
4 days	28 (12)
5 days	5 (3)
6 days	3 (1)
7 days	4 (2)
>7 days	56 (24)
Do not know	4 (2)

**Table 3:** Univariate analysis for risk factors of acute kidney injury OR, odds ratio; CI, confidence interval; UP, urine output; Hb, hemoglobin, RACHS, Risk Adjustment for Congenital Heart Surgery; CPB, cardiopulmonary bypass; ACC, aortic cross-clamp

Variables	Crude OR	95% CI	p-Value
Age (years)	1.0	0.99-1.03	0.50
<2	0.8	0.60-1.27	0.48
<5	0.7	0.52-1.08	0.12
Weight (kg)	1.0	0.99-1.03	0.47
Height (cm)	1.0	0.99-1.02	0.23
Gender	1.0	0.6-1.4	0.84
Preoperative UP (mL/kg/minutes)	1.1	0.83-1.51	0.44
Preoperative Hb (gm/dL)	1.0	0.99-1.12	0.05
Prior operation	0.5	0.25-1.28	0.12
RACHS-1 risk category 1	11.3	1.30-99.03	0.02
RACHS-1 risk category 2	5.7	0.68-47.79	0.10
RACHS-1 risk category 3	3.3	0.37-30.79	0.28
Cyanotic	1.3	0.9-1.83	0.23
Single ventricle	0.5	0.2-1.35	0.26
CPB time (minutes)	1.0	1.00-1.04	0.00
Prolonged CPB time (>120 minutes)	3.0	1.53-3.42	0.00
ACC time (minutes)	1.0	1.00-1.02	0.00
Multiple CPB	4.0	1.4-11.0	0.00
Multiple ACC	4.5	1.2-16.0	0.02
Modified ultra-filtrate	0.5	0.3-0.75	0.00
Need of blood transfusion	1.8	0.83-4.23	0.15
On arrival high inotropic score	1.0	1.00-1.06	0.01
On-arrival hypotension	1.4	0.6-3.10	0.34
Use of nephrotoxic antibiotics	1.9	0.9-3.67	0.05

**Table 4:** Multivariate analysis: risk adjusted for acute kidney injury OR, odds ratio; CI, confidence interval; CPB, cardiopulmonary bypass; Hb, hemoglobin

Variable	Adjusted OR	95% CI	p-Value
Prolonged CPB time (>120 minutes)	1.87	1.22, 2.88	0.00
Preoperative Hb (>16 gm/dL)	1.80	1.16, 2,78	0.00

## Discussion

Our study reported an overall frequency of AKI of approximately 28% after congenital heart surgery, without any association with mortality and morbidity. The frequency was lower than earlier reports, which may be due to our excluding the neonatal population in which the risk was highest. Ueno et al. also used m-KIDGO criteria and found 37.5% AKI in the infant population [11]. Another study by Sethi et al. used the AKIN criteria, as well as serum creatinine and baseline renal functions to define AKI based on the Schwartz formula [12]. This study did not use urine output due to the use of diuretics postoperatively. They reported an AKI prevalence of 9.6%, and most patients had stage I AKI. All patients with AKI recovered their kidney function at the time of discharge, with normal blood pressure. However, their study did not define the postoperative recovery course of their patients, although other studies did not have significant numbers of stage III AKI patients with spontaneous resolution [12,16,17]. We reported a slightly different observation: the majority (85%) of our patients were in stages II and III AKI according to the m-KDIGO classification, and 94% showed spontaneous resolution. Of the AKI cases, 6% worsened and required RRT; among the worsened cases, only 10 (4%) patients died. Twenty-seven percent of the patients were lost to follow-up. No further serum creatinine levels were used to document either complete resolution or the presence of residual AKI. Loss of follow-up and non-compliance to medical advice are also hidden factors for worse outcomes in low- to middle-income countries. To prevent bias and overdiagnosis of AKI, we used the m-KIDGO criteria, which required a minimum serum creatinine value of >0.5 mg/dL in order to be defined as AKI, as the recent literature supports the use of this definition for the diagnosis of AKI. Our age distribution also did not show a significant difference in the younger age group (i.e., nearly 54% of the patients younger than five years old). Of the affected cases, 87% belonged to RACHS-1 and RACHS-2, and almost half (45%) had cyanotic heart disease, whereas other reports had the majority of their patients in RACHS-2 and RACHS-3 [11,14]. The majority (66%) of AKI patients were diagnosed on their first postoperative day, and 64% of patients had their peak serum creatinine level within 24 hours after AKI diagnosis, which is similar to previous reports [14]. This highlights the important fact that AKI after CHD surgery was a common but self-limiting problem in most of the cases, but it can be associated with morbidity and increased resource utilization [1,2].

On multivariate analysis, prolonged CPB time and high hemoglobin were found to be independent risk factors. Li et al. found an interesting linear relationship between increasing bypass time and increasing AKI [2]. The association between prolonged CPB time and high hemoglobin (>16 gm/dL) could be explained by the presence of cyanotic heart disease in almost half of our study population, and our study population was older compared with that in previous reports. Severe, prolonged hypoxia can possibly affect the kidneys preoperatively, which manifests in the postoperative period. Another possible explanation could be the possibility of reperfusion injury; therefore, high hemoglobin is just a marker of severe hypoxia, which, in turn, is associated with AKI.

Many of the patients in our study were not monitored through follow-up in the clinic with a repeat creatinine level, which is mandatory in patients who have developed AKI after CHD surgery to detect residual disease and development of chronic kidney disease. We cannot comment on patients who were lost to follow-up or were diagnosed as stage II or III AKI based on an increase in serum creatinine. However, this study is the first of its kind to have highlighted the hidden facts, clinical outcomes, and shortcomings related to AKI patients from a low- to middle-income country. AKI is best managed with early detection and appropriate treatment to prevent associated morbidity and chronic kidney disease. There is considerable variability in clinical practices. Clinical practice guidelines in the ﬁeld thus have the potential to reduce variations, improve outcomes, and reduce costs.

There are some limitations in this study that could be addressed in future research. First, we included both pediatric and adult populations with the intent to perform a subgroup analysis for risk of AKI; however, this analysis could not be conducted possibly due to the paucity of adult AKI cases. Secondly, this was a single-center study with limited follow-up data; hence, the management of our center cannot be generalized to the entire population. Thirdly, there was a vast range of different cardiac surgeries, which we grouped into cyanotic, acyanotic, and single-ventricle heart diseases, and this made it difficult to correlate the type of surgery with the development of AKI. Lastly, we did not use urine output as a definition for AKI since many patients received diuretics, and this might have affected the accuracy of urine output as a variable for the definition of AKI.

## Conclusions

We found a high frequency of AKI after CHD surgery, and it was significantly associated with prolonged CPB time and high hemoglobin after congenital heart surgery. Though the majority of patients showed spontaneous resolution, not all were able to fulfill the complete resolution criteria. Therefore, proper clinical practice guidelines should be practiced in low- to middle-income countries to identify the risk factors and associations. This will make us more aware of the likelihood of developing AKI post-CHD surgery and thus allow earlier management of AKI, leading to reduced morbidity and mortality. We recommend that every postoperative patient be checked for AKI based on standard criteria. Regular follow-ups should be conducted for all AKI cases to determine long-term outcomes.
